# Trauma Symptoms, Life Satisfaction, and Perceived Happiness as Predictors of Mental Health in Displaced Children and Adolescents in Niger

**DOI:** 10.1007/s40653-026-00876-5

**Published:** 2026-04-18

**Authors:** Guido Veronese, Chiara Fiscone, Ihab Saleh, Ilaria Grazzani, Federica Cavazzoni

**Affiliations:** 1 Department of Human Sciences for Education “R. Massa”, University of Milano-Bicocca, Milan, Italy; 2 East Kent Hospitals University NHS Foundation Trust, Kent, United Kingdom

**Keywords:** Displaced children, Mental health, Life satisfaction, Trauma symptoms, Resilience, Socio-ecological model, Niger

## Abstract

This cross-sectional study examined relationships among trauma symptoms, mental health, life satisfaction, and perceived happiness in 265 displaced and refugee children in Niger (M age = 11.06). Guided by a socio-ecological and resilience-informed framework, the study explored both risk and protective factors. Results showed that life satisfaction was the strongest predictor of mental health, followed by perceived happiness, while trauma symptoms were not significant predictors. Unexpected findings included a negative association between life satisfaction and happiness and better outcomes among non-educated children, suggesting important cultural and contextual influences. The findings support strength-based, culturally sensitive interventions that prioritize relational and school-based resources to promote resilience.

## Introduction

Mental health is increasingly recognized as a multidimensional determinant of child development, especially in contexts of displacement, chronic violence, and structural deprivation. According to the World Health Organization (2004), mental health comprises a state of well-being in which individuals realize their potential, cope with normal life stressors, and positively engage with their communities. For conflict-affected children, mental health implies more than the absence of pathology. It also includes subjective well-being, emotional resilience, and the capacity to form and sustain meaningful social relationships (Masten, 2014; Tol et al., 2013; Veronese et al., 2019).

Despite this broad understanding, humanitarian responses and academic research have often been trauma-focused, focusing heavily on pathological outcomes—PTSD, depression, anxiety—and negating children’s agency, strengths, and contextual healing potential (Betancourt & Khan, 2008). Exposure to violence, displacement, and family separation constitutes a profound risk. However, longitudinal and cross-sectional research indicates that a substantial proportion of children do not develop enduring psychological disorders. For example, in a meta-analysis of forcibly displaced youth, ~ 80% showed psychological difficulties, but resilience-enhancing interventions—especially those delivered in non-clinical, community-based settings—demonstrated measurable gains in well-being and coping even without significant reductions in psychiatric symptoms (Hermosilla et al., 2021).

A comprehensive socio-ecological review encompassing over 60 studies published between 2010 and 2020 found that family cohesion, school connectedness, and peer support are robust protective factors across displacement contexts, acting at the individual, family, community, and cultural levels (Scharpf et al., 2021). Longitudinal data further emphasize that many children in conflict-affected low- and middle-income countries actually demonstrate recovery trajectories, with declines in PTSD, anxiety, and functional impairment over time, and increases in social support—even absent formal clinical intervention (34.6% improved over six months in one study) (Purgato et al., 2020).

We integrate war-related trauma and daily stressors within a socio-ecological perspective, emphasizing how contextual protective factors shape mental health outcomes (Panter-Brick & Eggerman, 2012; Fazel et al., 2012). The present study is grounded in socio-ecological theory and resilience science. Within this perspective, child mental health results from dynamic interactions between individual experiences and multiple contextual levels, including family, peers, school, community, and broader structural conditions. Psychological adjustment is shaped not only by reported post-traumatic symptoms, but also by ongoing daily stressors and the availability of protective psychosocial resources. Accordingly, trauma symptoms are conceptualized as key risk factors, whereas life satisfaction and perceived happiness are examined as indicators of resilience-related processes linked to social relationships, school engagement, and subjective well-being. In the present study, trauma symptoms represent a core risk factor associated with exposure to violence and displacement-related stressors. Trauma-related symptoms were assessed using the Children’s Revised Impact of Event Scale (CRIES-8), a widely used screening instrument designed to capture core post-traumatic stress responses (intrusion and avoidance) in children exposed to potentially traumatic events. In humanitarian and conflict-affected settings, detailed trauma exposure checklists are not always feasible or ethically appropriate. The CRIES-8 is therefore frequently used, as it assesses children’s subjective psychological responses without requiring explicit recounting of traumatic experiences. Consistent with this framework, the study hypotheses are derived from a dual focus on vulnerability and protection, examining how trauma-related symptoms and positive psychosocial resources jointly contribute to mental health outcomes among displaced and refugee children.

Consequently, the study does not include a direct measure of objective trauma exposure, but rather assesses children’s psychological responses to potentially traumatic events. Interpretations regarding “trauma levels” therefore refer strictly to symptom severity and not to the number or type of traumatic events experienced.

For instance, research among Syrian refugee children found that relational capacities and pro-social behavior are key protective outcomes that may mitigate trauma more effectively than symptom-focused therapy alone, suggesting the value of salutogenic, systems-oriented psychosocial interventions (Veronese et al., 2021).

The Sahel region exemplifies the urgent need for such nuanced perspectives. With recurrent violence, environmental stressors, and political fragility, the Sahel—especially Niger’s Diffa region—experiences large-scale displacement linked to Boko Haram and broader instability. By early 2021, Niger hosted over 313,000 internally displaced persons and more than 235,000 refugees, most of whom were children facing overlapping crises of violence, family separation, poverty, and limited schooling access (UNICEF, 2021). It is estimated that up to 80% of these children may develop psychological trauma, yet current mental health interventions remain largely under-prioritized (UNHCR, 2024).

Moreover, a substantial literature on child mental health in conflict-affected settings exists in contexts such as Syria, South Sudan, and Palestine, whereas research from Niger and the broader Sahel is still relatively scarce (Miller & Rasmussen, 2010; Veronese et al., 2022).

Despite the growing body of research on the mental health of children affected by armed conflict and displacement, empirical evidence from the Sahel region remains limited. In particular, Niger—and the Diffa region specifically—has received relatively little attention in peer-reviewed psychological research, despite hosting large populations of internally displaced and refugee children exposed to prolonged violence, poverty, and educational disruption. As a result, current knowledge on child mental health in humanitarian settings is still disproportionately informed by a small number of contexts, leaving important geographical and cultural gaps in the evidence base.

Within a socio-ecological and resilience-informed framework, child mental health is conceptualized as a multidimensional outcome reflecting both psychological functioning and subjective well-being (Rees & Main, 2016). In the present study, trauma symptoms represent a core risk factor associated with exposure to violence and displacement-related stressors. Life satisfaction is understood as a cognitive–evaluative component of well-being, reflecting children’s appraisal of key life domains such as peer relationships, school experiences, and self-perception, which have been identified as central protective resources in conflict-affected contexts (Huebner et al., 2013). Perceived happiness, by contrast, captures a more immediate affective dimension of well-being, reflecting children’s global emotional state (Holder, 2012). In humanitarian and low-literacy contexts, perceived happiness is often assessed using brief, visually based measures that minimize cognitive and linguistic demands (Veenhoven, 2017). To enhance conceptual clarity, the present study distinguishes between related but non-equivalent constructs. Mental health is operationalized as a multidimensional construct encompassing psychological functioning (CHQ), whereas psychological well-being refers specifically to the emotional and self-evaluative subdimensions of this broader construct. Life satisfaction is conceptualized as a cognitive–evaluative appraisal of domain-specific conditions (e.g., peers, school, self), while perceived happiness captures an immediate affective state. Although interrelated, these constructs are not assumed to be interchangeable, particularly in low-resource and non-Western contexts where their empirical associations may diverge.

Accordingly, the present study operationalizes perceived happiness using a Face Scale, which has been shown to be developmentally appropriate, culturally flexible, and particularly suitable for use with young children and vulnerable populations in non-Western and crisis-affected settings. Drawing on resilience science, both life satisfaction and perceived happiness are conceptualized as indicators of adaptive processes that may buffer the negative impact of trauma and contribute to positive mental health outcomes (Cohn et al., 2009). Accordingly, the study examines how trauma-related distress and positive psychosocial resources are theoretically expected to interact in shaping mental health among displaced and refugee children.

The present study focuses on the psychosocial well-being of displaced and refugee children in Niger, considering both trauma-related distress and positive dimensions of mental health: life satisfaction, perceived happiness, and social belonging. Guided by socio-ecological theory and resilience science, the study examines how trauma-related symptoms, protective social variables (e.g. peer cohesion, educational access), and sociodemographic and cultural factors jointly shape mental health outcomes. In doing so, it aligns with global calls for interventions that promote dignity, agency, and collective recovery, rather than those focused exclusively on pathology (IASC, 2007; United Nations Convention on the Rights of the Child, 1989; Ungar, 2011).

## The Study

This cross-sectional quantitative study investigates the interrelationships among trauma-related symptoms, mental health, life satisfaction, and perceived happiness in a sample of internally displaced and refugee children and adolescents living in Niger. Rooted in a socio-ecological and resilience-informed framework, the study aims to examine on both the risk and protective factors that shape psychological well-being in youth affected by displacement and conflict.

The first objective is to examine group differences in trauma symptoms, mental health outcomes, life satisfaction, and perceived happiness across key sociodemographic variables—namely gender, age group, displacement status (refugee vs. internally displaced), level of education, and ethnic background. These factors are known to influence children’s psychological adjustment in humanitarian settings and may moderate the impact of trauma or access to protective resources.

The second objective is to explore the predictive relationships among trauma, life satisfaction, and happiness in relation to mental health outcomes. Specifically, we aim to assess the extent to which trauma (as a risk factor) and life satisfaction and happiness (as protective factors) explain variability in psychological well-being, conceptualized as a continuum ranging from distress to resilience.

Empirical research in conflict-affected and displaced populations consistently demonstrates that exposure to war-related violence and chronic stressors is associated with elevated trauma-related symptoms and poorer mental health outcomes, particularly among children facing cumulative and prolonged adversity (Masten, 2014; Tol et al., 2013). At the same time, socio-ecological and resilience-oriented studies highlight that psychological adjustment is shaped by differential exposure to risk and protection across developmental stages and social contexts, including age, gender, access to education, and displacement-related conditions (Fazel et al., 2012; Betancourt et al., 2013; Scharpf et al., 2021). Moreover, positive dimensions of subjective well-being—such as life satisfaction and perceived happiness—have been shown to function as protective resources that support coping and adaptation in adverse environments, although their effects may vary across cultural and contextual settings (Huebner et al., 2013; Veronese et al., 2019). Drawing on this body of evidence, the present study formulates theory-driven, directional hypotheses regarding the associations between trauma-related symptoms, sociodemographic characteristics, positive psychosocial resources, and mental health outcomes.

Based on the socio-ecological and resilience-informed framework outlined above, and supported by existing empirical literature, we propose the following hypotheses:

### H1

There will be statistically significant differences in trauma-related symptoms, mental health outcomes, life satisfaction, and perceived happiness across sociodemographic subgroups (e.g., gender, age, displacement status, educational level, and ethnic identity), with older age, female gender, and greater displacement-related disadvantage expected to be associated with higher trauma-related symptoms and lower levels of subjective well-being. This hypothesis is supported by evidence that demographic and contextual variables systematically influence psychological outcomes in displaced populations (Fazel et al., 2012; Betancourt et al., 2013).

### H2

Trauma-related symptoms are expected to be negatively associated with mental health outcomes; however, within a socio-ecological and resilience-informed framework, this association may be attenuated when protective psychosocial resources are considered. (Tol et al., 2013; Masten, 2014; Çelik & Özkan, 2024).

### H3

Perceived happiness is expected to be associated with mental health outcomes, reflecting its role as an affective component of well-being; however, its relationship with specific dimensions of psychological functioning may vary depending on contextual and cultural factors. (Lyubomirsky et al., 2005; Suldo et al., 2006).

### H4

Life satisfaction—particularly in relational and school-related domains—is expected to positively predict mental health outcomes, indicating that greater satisfaction with key life domains is associated with improved psychological functioning (Veronese et al., 2019; Panter-Brick & Eggerman, 2012).

Together, these hypotheses reflect a dual emphasis on vulnerability and resilience, allowing for a nuanced understanding of how mental health is shaped not only by the presence of risk factors such as trauma, but also by the presence of positive psychosocial resources that promote adjustment and well-being in the face of adversity.

### The Study Background

This study was conducted in the Diffa region of the Republic of Niger, a southeastern area within the Lake Chad Basin that has been severely affected by displacement, conflict, and poverty. Niger, a landlocked country in West Africa, has experienced chronic political instability since its independence in 1960, including military coups, terrorism, and governance crises—most recently, the 2023 overthrow of President Mohamed Bazoum (ISPI, 2023). In addition to conflict and poverty, Niger’s role as a major transit hub for African and transcontinental migration has further exacerbated humanitarian challenges such as displacement and insecurity (Amato & Iocchi, 2019). The country is ethnically diverse, with the Hausa comprising 53% of the population, alongside Arab, Kanuri, and Fulani communities (Amato & Iocchi, 2019).

Children in Niger endure some of the harshest living conditions globally, facing widespread exposure to violence, forced displacement, and structural instability. The country ranks among the lowest in development indicators: it has a fertility rate of seven children per woman, a life expectancy of 55.9 years, and an infant mortality rate close to 80%. It also has one of the youngest populations in the world, with a median age of 15.4 years, and limited access to education, with national literacy rates at only 31% as of 2012 (Amato & Iocchi, 2019; MAECI, 2024). Approximately 2.5 million children are out of school, and only 19% of rural girls complete primary education, due to factors such as poverty, child labor, and insecurity (MAECI, 2024).

In Diffa specifically, the ongoing humanitarian crisis has resulted in the closure of over 300 schools, affecting at least 22,000 children in 2020 alone (UNICEF, 2021). Since 2015, more than 100,000 children aged 4 to 17—over half of them girls—have been denied schooling because of displacement and conflict (DREC, 2019). Currently, the region hosts over 230,000 internally displaced persons and more than 300,000 refugees (UNICEF, 2021). Alarmingly, the situation continues to deteriorate: the number of displaced children in Mali, Burkina Faso, and Niger increased from 321,000 in 2019 to approximately 1.8 million by 2024, the vast majority of whom remain within their countries (Save the Children, 2024).

## Methods

### Participants

A total of 265 minors aged between 5 and 18 years (M = 11.06, SD = 2.77) participated in the study, of whom 46.8% were male. Participants were recruited through purposive convenience sampling, in collaboration with an international non-governmental organization (INGO) providing shelter and support to displaced populations in Niger’s Diffa region.

Inclusion criteria required that participants be under the age of 18 and have a parent or legal guardian who had received support from the partnering international NGO. Eligibility for participation was based on documented forced displacement due to armed conflict or terrorist violence within the preceding 12 months, as confirmed through caregivers’ reports and the partnering humanitarian organization; therefore, all participants were assumed to have been exposed to conflict-related stressors, although no separate trauma-related symptoms checklist was administered. Participants must have experienced forced internal displacement or held refugee status within the preceding 12 months as a result of armed conflict or terrorist violence originating in neighboring Sub-Saharan countries. Additionally, participants were required to be residing in a refugee camp managed by UNHCR or an affiliated NGO and to be accompanied by a parent or legal guardian at the time of data collection.

Exclusion criteria included unaccompanied minor status, lack of direct exposure to armed conflict, non–Sub-Saharan origin, and the presence of severe mental health disorders in either the child or their caregiver.

### Instruments and Procedures

This study employed a set of validated psychometric instruments to assess the core constructs of interest in a sample of minors in Niger. The assessment focused on four key domains: mental health, trauma symptoms, overall happiness, and life satisfaction. Data were collected between January and April 2024 in a safe and controlled environment designed to ensure participants’ privacy and protect their rights. Parents or legal guardians were present throughout the assessment to provide emotional support and reassurance to the children.

Data collection was facilitated by trained personnel from a local non-governmental organization, who obtained informed consent from parents or guardians using clear, age-appropriate language and emphasized the voluntary nature of participation. The instruments were administered in paper-and-pencil format and were available in both English and French to accommodate participants’ linguistic and cultural contexts.

A detailed description of the measures used in the study is provided below.

#### Child Health Questionnaire (CHQ)

The CHQ is a standardized instrument designed to assess overall health—both physical and mental—in children aged 5 to 18 years (QualityMetric, 2025). Available in more than 78 languages (Landgraf, 2020), it covers over 90 clinical conditions across 14 health domains. In this study, a short version adapted by Veronese and Pepe (2017) was administered to parents. This version consists of six items distributed across two subscales: *psychological well-being* (e.g., “How often during the day does your child cry?”) and *self-esteem* (e.g., “Do you think your child is satisfied with their academic abilities?”). Items are rated on a 5-point Likert scale ranging from 0 (“not at all”) to 4 (“extremely”), with reverse-scored items appropriately recoded (i.e., 0 becomes 4, 1 becomes 3, etc.). In this sample, the instrument demonstrated acceptable internal consistency (Cronbach’s alpha = 0.77).

#### Children’s Revised Impact of Event Scale (CRIES-8)

The CRIES-8 (Children and War Foundation, 1998; Perrin et al., 2005) is a brief, easy-to-administer tool for assessing the psychological impact of traumatic events in children aged 8 years and older. It consists of eight items equally divided between two subscales: *intrusion* (e.g., “Do you have strong feelings about the event?”) and *avoidance* (e.g., “Do you try not to think about the event?”). In this study, the version adapted by Veronese and Pepe (2017) was used, with a 5-point response scale ranging from 0 to 4. Children completed the measure themselves. Scores were summed for each subscale, with no reverse-coded items. The internal consistency of the CRIES-8 in this sample was excellent (Cronbach’s alpha = 0.94).

#### Multidimensional Students’ Life Satisfaction Scale – Short Version (MSLSS)

Originally developed by Huebner (1994), the MSLSS assesses life satisfaction across five domains. This study employed a short, culturally adapted version (Veronese & Pepe, 2017), focusing on three domains: *friends* (e.g., “Are your friends kind to you?”), *school-related support activities* (e.g., “Are school activities interesting?”), and *self* (e.g., “Are you a likable person?”). The 20-item scale was completed directly by the children, with items rated on a 5-point Likert scale (0 to 4). Reverse-coded items were adjusted according to standard scoring procedures. This scale showed strong internal reliability in the current sample (Cronbach’s alpha = 0.87).

#### Face Scale (FS)

The Face Scale (Andrews & Withey, 1976) is a simple, widely used instrument for measuring perceived overall happiness. It consists of a single item: a row of seven faces ranging from extreme sadness to extreme happiness. Children are asked to select the face that best represents their typical mood (Veronese et al., 2012). The FS is particularly suitable for young children (from age 3) because it allows them to express emotions nonverbally through visual representation.

Given the multi-ethnic composition of the sample, the issue of measurement equivalence was carefully considered. However, formal measurement invariance testing across ethnic groups was not conducted, primarily due to sample size constraints within specific subgroups and the exploratory nature of the study. All instruments used have been widely applied in diverse cultural and humanitarian settings, including African and non-Western contexts, supporting their cross-cultural applicability.

### Data Analysis

All statistical analyses were conducted using SPSS software (Version 29.00.). Prior to inferential testing, assumptions of normality were assessed by examining skewness and kurtosis values, which were considered acceptable if within the range of −2 to + 2 (George & Mallery, 2009). Internal consistency of each scale was evaluated using Cronbach’s alpha. Participants with more than 20% missing responses on any of the administered measures were excluded from the final analyses, as their data were deemed insufficient for reliable interpretation. Descriptive statistics were first computed to summarize the sample’s sociodemographic characteristics, including gender, age group (categorized according to WHO standards by Ahmad et al., 2001: children 5–9, preadolescents 10–14, adolescents 15–18), displacement status, ethnic background, and educational level. Additional descriptive analyses were conducted to calculate means, standard deviations, and minimum/maximum values for each scale and subscale. Group differences based on sociodemographic variables were examined using independent samples t-tests (for gender and educational level) and multivariate analysis of variance (MANOVA) for age group, displacement status, and ethnicity. Tukey post hoc tests were applied to identify specific between-group differences, using a significance threshold of α = 0.05.

To explore relationships among psychological constructs, Pearson correlation coefficients were computed for total scores and subdimensions of each measure. Finally, three multiple linear regression analyses were conducted to investigate the predictive role of trauma (CRIES), life satisfaction (MSLSS), and perceived happiness (FS) as independent variables, on mental health outcomes (CHQ), both at the level of the total score and its subcomponents. This approach allowed for a more nuanced understanding of how these psychosocial factors relate to children’s and adolescents’ mental health within the context of displacement.

### Ethical Considerations

Before data collection began, the INGO provided both participants and their parents with comprehensive information regarding the study’s aims and procedures. Parents were clearly informed of their right to refuse their child’s involvement, and children were assured that participation was voluntary, they could skip any question or withdraw from the interview at any point without consequence. Following this briefing, informed consent was obtained from parents, and children provided their assent, with full assurance of anonymity and confidentiality.

Throughout the data collection phase, local psychologists were present to offer debriefing and individual support to any participant experiencing emotional discomfort. The study received ethical approval from the Ethics Committee of Niamey’s General Hospital (Niger). All procedures complied with the ethical guidelines set forth in the 2013 revision of the Declaration of Helsinki and the 2020 ethical standards and code of conduct of the American Psychological Association (APA).

## Results

### Descriptive Statistics

A total of 265 minors aged between 5 and 18 years (M = 11.06; SD = 2.77) participated in the study, with 46.8% identifying as male. Based on WHO age group classifications, the sample comprised 23% children (5–9 years), 57% preadolescents (10–14 years), and 10.2% adolescents (15–18 years).

In terms of displacement status, 54.7% of participants were internally displaced, 29.1% were classified as refugees, and 6.4% as asylum seekers. Educational attainment was generally low: 79.2% of participants had never attended school, while only 10.9% reported having received some form of formal education. From an ethnic standpoint, the sample was predominantly Kanuri (35.5%), followed by Peul (18.1%), Arab (17.7%), and Hausa (15.1%). A small proportion (3.8%) identified with other minoritarian ethnic groups.

For clarity, higher scores on the CHQ indicate better mental health, higher CRIES scores indicate greater trauma-related symptoms, higher MSLSS scores indicate greater life satisfaction, and higher FS scores indicate greater perceived happiness.

Table 1 provides descriptive statistics for the total scores and subdimensions of the study variables (MSLSS, CRIES, CHQ, and FS).

**Table 1:** Descriptive statistics of study variables

Life satisfaction	Min	Max	Mean	SD	Skewness	Kurtosis
	32.00	80.00	63.89	7.29	0.157	0.314
Friends	0	24	17.48	2.71	0.150	0.298
School	12	28	23.68	2.84	0.157	0.314
Self	14	28	22.93	3.46	0.150	0.298
Trauma symptoms	0.00	32.00	7.45	9.20	0.157	0.314
Intrusion	0	16	2.87	4.25	0.156	0.310
Avoidance	0	16	4.60	5.57	0.157	0.313
Mental health	4.00	24.00	19.21	2.91	0.150	0.298
Self-confidence	0	12	8.98	1.87	0.150	0.298
Psychological well-being	0	12	10.23	2.08	0.150	0.298
Overall Happiness	1.00	7.00	2.70	0.99	0.150	0.298

Higher scores indicate greater levels of the measured construct (i.e., higher CHQ = better mental health; higher CRIES = greater trauma symptoms; higher MSLSS = higher life satisfaction; higher FS = higher perceived happiness)

### Mean differences

Starting with trauma symptoms, the MANOVA revealed significant group differences based on age (Wilks’ Λ = 0.818; F(16, 414) = 2.738; *p* <.001), displacement status (Wilks’ Λ = 0.883; F(16, 414) = 1.665; *p* =.05), and ethnic group (Wilks’ Λ = 0.616; F(32, 757) = 3.328; *p* <.001). Follow-up analyses indicated significant differences in the CRIES total score (F = 6.947; *p* =.001; ηp² = 0.061), intrusion (F = 4.624; *p* =.011; ηp² = 0.041), and avoidance (F = 7.371; *p* <.001; ηp² = 0.064) across age groups, with adolescents reporting the highest levels of trauma symptoms (M = 11.76), followed by preadolescents (M = 6.77) and children (M = 4.05). In terms of ethnicity, significant differences emerged for the trauma total score (F = 2.862; *p* =.024; ηp² = 0.051), intrusion (F = 2.483; *p* =.045; ηp² = 0.045), and avoidance (F = 2.943; *p* =.021; ηp² = 0.053). The Hausa group showed the highest levels of trauma (M = 10.02), compared to Peul (M = 8.43), Kanuri (M = 5.09), Arabs (M = 5.32), and other minoritarian groups (M = 5.60). Post hoc Tukey tests confirmed significant differences in total trauma scores between children and adolescents (*p* <.001) and between preadolescents and adolescents (*p* =.02). For ethnicity, significant differences were observed between Hausa and Kanuri in both total trauma (*p* =.031) and avoidance (*p* =.014), while no significant pairwise differences emerged for intrusion. These mean-level differences should be interpreted cautiously, as formal tests of measurement invariance across ethnic groups were not conducted.

Turning to mental health, MANOVA showed significant effects of both displacement status (F = 4.358; *p* =.014; ηp² = 0.039) and ethnic group (F = 4.718; *p* =.001; ηp² = 0.082) on the CHQ total score, as well as on the self-confidence subscale. Internally displaced children reported higher mental health scores (M = 19.64) than refugees (M = 19.13) and asylum seekers (M = 17.73. Among ethnicities, the Peul scored highest in both mental health (M = 20.50) and self-confidence (M = 9.66), followed by Kanuri (M = 19.50; M = 9.05), Arabs (M = 18.42; M = 8.47), and Hausa (M = 18.69; M = 8.54). T-tests revealed significant differences by educational level, with higher scores among educated participants for overall mental health (M = 20.68 vs. 19.17; t = −3.044, *p* =.003, d = −0.603) and psychological well-being (M = 11.34 vs. 10.28; t = −3.187; *p* =.002; d = −0.631). However, the negative direction of the test statistics suggests that non-educated participants actually reported better psychological outcomes. Post hoc comparisons confirmed significant differences between displaced and asylum seekers groups, and between Peul vs. Arabs and Peul vs. Hausa.

Focusing next on life satisfaction, ethnic background had a strong effect on the MSLSS total score (F = 16.25; *p* <.001, ηp² = 0.235) and its three subdomains: friends (F = 7.393; *p* <.001, ηp² = 0.122), school (F = 6.209; *p* <.001, ηp² = 0.105), and self (F = 19.943; *p* <.001, ηp² = 0.273), all indicating large effect sizes. The Peul group consistently reported the highest life satisfaction (M = 68.25), followed by Kanuri (M = 65.75), Hausa (M = 61.02), other minoritarian groups (M = 60.00), and Arabs (M = 58.80).Post hoc analyses confirmed significant group differences, particularly between Peul vs. Arabs and Peul vs. Hausa. T-tests also showed that non-educated children reported higher total life satisfaction (M = 64.79 vs. 61.20; t = −2.33; *p* =.021, d = −0.462) and notably higher satisfaction in the “self” domain (M = 22.50 vs. 20.00; t = −4.128; *p* <.001, d = −0.624).

Finally, regarding overall happiness, MANOVA indicated significant effects of age group (F = 5.222; *p* =.006, ηp² = 0.047) and ethnicity (F = 5.716; *p* <.001, ηp² = 0.097). Preadolescents reported the highest happiness (M = 5.28), followed by children (M = 4.96) and adolescents (M = 4.85). Among ethnic groups, the Peul again showed the highest scores (M = 5.50), ahead of Kanuri (M = 5.10), others (M = 4.92), Hausa (M = 4.89), and Arabs (M = 4.74). Post hoc comparisons revealed significant differences between children and preadolescents (*p* =.004), and among ethnic groups, particularly Peul vs. Arabs, Peul vs. Hausa, and Peul vs. other minoritarian groups. A significant difference also emerged by educational level, with non-educated children reporting higher happiness (M = 5.20) than their educated peers (M = 4.83; t = 2.025; *p* =.044, d = 0.401).

According to conventional benchmarks (ηp² ≈ 0.01 small, 0.06 medium, 0.14 large), most observed effects ranged from small to medium magnitude, with the exception of ethnic differences in life satisfaction domains, which approached large effect sizes.

Given the absence of formal measurement invariance testing, these differences should be interpreted as observed mean variations rather than definitive cultural differences in underlying constructs.

Correlational analyses revealed several significant relationships among the total scores and subdimensions of the psychological scales. Life satisfaction was moderately and positively associated with mental health overall (*r* =.366; *p* <.001), and with its subcomponents: self-confidence (*r* =.351; *p* <.001) and psychological well-being (*r* =.223; *p* <.001). Within life satisfaction, all subdomains, friends, school, and self-perception, were positively correlated with one another and with mental health. However, life satisfaction showed a strong negative correlation with happiness (*r* = –.528; *p* <.001), particularly in the subdimensions of friends (*r* = –.427), school (*r* = –.314), and self-perception (*r* = –.499), all *p* <.001. Mental health was positively correlated not only with the total and subscale scores of life satisfaction, but also with happiness: negatively through self-confidence (*r* = –.158; *p* =.010), and positively through psychological well-being (*r* =.126; *p* =.040). Self-confidence was also associated with higher satisfaction in friendships (*r* =.278), school (*r* =.264), and self-perception (*r* =.321), all *p* <.001. Overall happiness demonstrated a consistent pattern of negative correlations with trauma (*r* = –.161; *p* =.013), particularly with the intrusion (*r* = –.154; *p* =.016) and avoidance (*r* = –.158, *p* =.014) subdimensions. It was also inversely correlated with self-confidence and life satisfaction across all domains. Interestingly, trauma symptoms, while generally unrelated or inversely related to well-being indicators, showed a weak positive correlation with the self-perception domain of life satisfaction (*r* =.133; *p* =.040) and, more specifically, between intrusion and both self-perception (*r* =.162; *p* =.011) and self-confidence (*r* =.142; *p* =.027). Finally, the school domain of life satisfaction was positively associated with both mental health (*r* =.298; *p* <.001) and psychological well-being (*r* =.201; *p* =.002), reinforcing the relevance of educational experience to emotional adjustment.

### Linear Regression

Multiple linear regression analyses revealed several significant findings. In the first model (Table 2), the independent variables—happiness, trauma, and life satisfaction—jointly accounted for 15% of the variance in overall mental health outcomes [R² = 0.148, F(3, 209) = 12.102; *p* <.001]. Among the predictors, life satisfaction emerged as the strongest positive predictor (b = 0.147, β = 0.435, t = 5.973, *p* <.001), followed by happiness (b = 0.624, β = 0.239, t = 3.28; *p* =.001). Trauma, however, was not a significant predictor.

**Table 2:** Multiple linear regression analysis predicting Mental Health (CHQ) from Happiness (FS), Life Satisfaction (MSLSS), and Trauma symptoms (CRIES)

	B	SE	β	t	Sig.
Happiness	0.624	0.190	0.239	3.281	0.001
Trauma symptoms	0.010	0.017	0.038	0.586	0.558
Life satisfaction	0.147	0.025	0.435	5.973	< 0.001

*β *standardized regression coefficient, *CHQ *Child Health Questionnaire, *CRIES *Children’s Revised Impact of Event Scale, *MSLSS *Multidimensional Students’ Life Satisfaction Scale, *FS * Face Scale

A second model examined predictors of psychological well-being, incorporating the subdimensions of trauma, happiness, and life satisfaction (Table 3). This model explained 9% of the variance [R² = 0.091, F(6, 210) = 3.516; *p* =.002]. Notably, happiness emerged as a significant negative predictor (b = –0.398, β = –0.214, t = − 2.672; *p* =.008), while the “friends” subdimension of life satisfaction was a significant positive predictor (b = 0.139, β = 0.210, t = 2.386; *p* =.018). Other subdimensions, school, self-perception, intrusion, and avoidance, were not significant.

**Table 3:** Multiple linear regression analysis predicting Psychological Well-being (CHQ subscale) from the subdimensions of Trauma symptoms (CRIES), Happiness (FS), and Life Satisfaction (MSLSS)

		B	SE	β	t	Sig.
Overall Happiness		− 0.398	0.149	− 0.214	−2.672	0.008
Life Satisfaction	Friends	0.139	0.058	0.210	2.386	0.018
	Self	0.046	0.049	0.089	0.939	0.349
	School	0.069	0.046	0.115	1.480	0.140
Trauma symptoms	Intrusion	− 0.082	0.046	− 0.188	−1.797	0.074
	Avoidance	0.040	0.032	0.129	1.251	0.212

*β *standardized regression coefficient, *CHQ *Child Health Questionnaire, *CRIES *Children’s Revised Impact of Event Scale, *MSLSS *Multidimensional Students’ Life Satisfaction Scale, *FS *Face Scale

The third model (Table 4), predicting self-confidence, was also statistically significant, explaining 16% of the variance [R² = 0.160, F(6, 210) = 6.650; *p* <.001]. Two significant predictors emerged: the “school” subdimension of life satisfaction (b = 0.091, β = 0.172, t = 2.311; *p* =.022) and the “friends” subdimension (b = 0.126, β = 0.217, t = 2.560; *p* =.011). No other predictors reached significance.

**Table 4:** Multiple linear regression analysis predicting Self-confidence (CHQ subscale) from the subdimensions of Trauma (CRIES), Happiness (FS), and Life Satisfaction (MSLSS)

		B	SE	β	t	Sig.
Overall Happiness		− 0.030	0.126	− 0.019	− 0.241	0.809
Life Satisfaction	Friends	0.126	0.049	0.217	2.560	0.011
	Self	0.037	0.041	0.082	0.906	0.366
	School	0.091	0.039	0.172	2.311	0.022
Trauma symptoms	Intrusion	0.043	0.038	0.112	1.109	0.269
	Avoidance	− 0.003	0.027	− 0.009	− 0.096	0.924

*β *standardized regression coefficient, *CHQ *Child Health Questionnaire, *CRIES *Children’s Revised Impact of Event Scale, *MSLSS *Multidimensional Students’ Life Satisfaction Scale, *FS *Face Scale

## Discussion

The present study examined the mental health of internally displaced and refugee children and adolescents living in the Diffa region of Niger, a context characterized by conflict, displacement, and structural instability. The first objective was to assess differences in trauma-related symptoms, mental health, life satisfaction, and perceived happiness across key sociodemographic variables, including gender, age group, displacement status, educational level, and ethnic background. The second objective was to explore whether distinct dimensions of psychological functioning—namely trauma-related distress, cognitive-evaluative well-being (life satisfaction), and affective well-being (happiness)—predicted mental health outcomes. By addressing these aims, the study contributes context-specific evidence to inform culturally sensitive and resilience-oriented interventions in humanitarian settings.

With respect to the study hypotheses, not all expectations were empirically confirmed. Specifically, H2, which posited a negative association between trauma-related symptoms and mental health, was not supported in the regression models. Similarly, H3 was only partially supported, as perceived happiness showed inconsistent associations depending on the outcome considered. While non-confirmation of hypotheses may appear counterintuitive, these findings are consistent with socio-ecological models suggesting that trauma exposure alone does not uniformly predict psychological outcomes when contextual protective factors are taken into account. These results therefore refine, rather than contradict, the theoretical framework guiding the study.

The findings indicate that life satisfaction, particularly in the domains of friendships and school engagement, emerged as the most robust predictor of both overall mental health and self-confidence. This result aligns with prior research emphasizing life satisfaction as a protective resource that supports psychological adaptation in conflict-affected populations (Veronese et al., 2017, 2019; Scharpf et al., 2021). Peer relationships and meaningful school experiences appear to provide structure, belonging, and relational safety within unstable environments. These findings reinforce evidence suggesting that school-based and peer-supported interventions can promote well-being even in the presence of ongoing adversity (Tol et al., 2011; IASC, 2007).

Perceived happiness showed a more complex pattern. While it was positively associated with overall mental health, it negatively predicted psychological well-being in regression analyses. This apparent contradiction may reflect the conceptual distinction between happiness as a short-term affective state and life satisfaction as a more stable evaluative component of well-being (Helliwell et al., 2023). The Face Scale captures global emotional expression through visual representation, whereas the MSLSS assesses structured evaluations of relational and school domains. In low-literacy humanitarian settings, children may interpret affective scales differently, and responses may reflect social display norms rather than stable psychological functioning (Speidel et al., 2021). The strong negative correlation between life satisfaction and happiness suggests potential measurement misalignment or contextual differences in how emotional states are expressed. Future research should examine whether scale directionality, valence perception, or culturally specific emotional norms influence these patterns. Importantly, such divergent or even contradictory associations are theoretically plausible within socio-ecological and cross-cultural frameworks. In low-literacy and non-Western settings, affective expressions of happiness may reflect situational, relational, or socially mediated states rather than stable indicators of psychological functioning. As such, discrepancies between affective and cognitive components of well-being should not be interpreted as inconsistencies, but rather as contextually grounded variations in how well-being is experienced and reported.

Contrary to initial expectations, trauma-related symptoms did not significantly predict mental health outcomes in the regression models. Rather than indicating limited exposure to violence, this finding suggests that self-reported trauma symptoms may not independently explain variance in mental health once protective psychosocial resources are considered. This pattern is consistent with socio-ecological models emphasizing the interaction between distress and contextual supports (Miller & Rasmussen, 2010; Masten, 2018; Tol et al., 2020). Similar findings from other humanitarian contexts show that trauma symptoms alone are often insufficient to explain variability in child adjustment when relational and environmental factors are taken into account (Bhutta et al., 2023; Panter-Brick et al., 2018; Veronese et al., 2021). These results support integrative approaches that examine both risk and protection.

Several unexpected patterns emerged. Non-educated children reported higher life satisfaction and happiness than educated peers. These paradoxical findings may reflect social desirability bias, culturally specific understandings of well-being, or differences in expectations across groups (Jordans et al., 2011; Summerfield, 2000). Educational settings in displacement contexts may also introduce stress related to instability, disrupted schooling, or performance demands (Tyrer & Fazel, 2014). Further research should explore how children experience both formal and informal learning environments under conditions of displacement.

Ethnic differences in observed well-being scores were also notable. Peul children reported higher life satisfaction, happiness, and mental health scores compared to other groups. However, these differences must be interpreted cautiously given the absence of formal measurement invariance testing. Observed variations may reflect differences in community structure, displacement experiences, or response styles rather than definitive cultural differences in underlying constructs. These findings highlight the importance of culturally sensitive programming that recognizes variation without overgeneralization (Betancourt, 2012; Scharpf et al., 2021).

Age differences were consistent with developmental expectations. Adolescents reported higher trauma-related symptoms and lower happiness compared to younger children. Developmental literature suggests that older youth accumulate greater exposure to stressors and face additional identity-related challenges (Masten, 2014; Otorkpa et al., 2024). Age-sensitive interventions that support adolescents’ agency and social connectedness are therefore particularly important.

Overall, the findings underscore the importance of prioritizing relational and educational domains in psychosocial programming. Life satisfaction, especially in the areas of friendships and school engagement, appears central to children’s psychological adjustment. Interventions embedded within community and school contexts may therefore offer sustainable pathways for promoting resilience (Tol et al., 2011; IASC, 2007).

By situating mental health within a socio-ecological framework, this study contributes to strength-based and context-sensitive approaches in global mental health research (Bartniczak et al., 2024; Betancourt et al., 2020; Tol et al., 2020; Theron, 2023). Evidence from underrepresented regions such as the Sahel remains limited, and the present findings respond to calls for research that foregrounds locally meaningful indicators of well-being alongside trauma-related distress.

## Limitations and Future Directions

This study has several limitations that should be acknowledged. First, its cross-sectional design precludes any inference of causality between levels of trauma symptoms, well-being, and mental health outcomes. Longitudinal research is needed to capture changes over time and to better understand the dynamic interplay between risk and protective factors (Tol et al., 2011).

Second, the reliance on self-report and parent-report measures may introduce response biases, such as social desirability or underreporting of distress, particularly in contexts where stigma surrounds mental health. Third, the potential cultural misalignment of Western-developed psychometric tools may limit the accuracy and validity of the findings, as such instruments may fail to fully capture locally meaningful expressions of suffering, resilience, and well-being (Kohrt et al., 2011, 2014). Moreover, although the study included children from multiple ethnic backgrounds, formal measurement invariance testing was not performed. As a result, mean-level comparisons across ethnic groups should be interpreted with caution, as observed differences may partly reflect cultural variations in response styles rather than true differences in underlying constructs. Finally, the use of convenience sampling within specific camps in the Diffa region limits the generalizability of the results to other displaced populations in Niger or the broader Sahel. In addition, participants were recruited through purposive convenience sampling within a single NGO-supported setting in Diffa. Children residing in non-supported camps, informal settlements, or host communities may experience different stressors and access to resources. Therefore, findings cannot be generalized to all displaced children in Niger or the broader Sahel region.

These limitations, however, are common in humanitarian research, where logistical, ethical, and contextual constraints often shape study design and implementation (Tol et al., 2011). Despite these challenges, the findings provide valuable insights and highlight critical areas for further investigation.

Future research should adopt longitudinal and mixed-methods designs, combining quantitative measures with qualitative interviews or ethnographic approaches to capture both causal pathways and the richness of children’s lived experiences. Incorporating participatory and child-centered methodologies, such as involving youth as co-researchers or advisors, can enhance the relevance, cultural validity, and ethical integrity of studies in these settings (Hart, 2008).

In addition, future studies should prioritize the development and validation of culturally grounded assessment tools, co-created with affected communities to ensure they reflect local conceptions of mental health, distress, and resilience. Such tools would mitigate the limitations of applying Western-centric instruments and improve the accuracy of findings (Kohrt et al., 2011, 2014). Research should also explore how structural factors—such as access to education, community cohesion, and gender norms—interact with individual-level resilience to shape psychosocial outcomes.

Moreover, future work could examine the effectiveness of interventions that explicitly target life satisfaction domains, particularly through school-based, peer-supported, and community-embedded strategies. Evaluating such programs through rigorous designs (e.g., randomized controlled trials) would provide stronger evidence for their impact and scalability in humanitarian contexts.

By addressing these limitations and pursuing these directions, future research can contribute to a deeper, more nuanced, and culturally appropriate understanding of displaced children’s mental health. This, in turn, can inform holistic, resilience-based interventions that emphasize not only the alleviation of distress but also the cultivation of hope, agency, and social recovery (Panter-Brick & Eggerman, 2012).

## Conclusion

In conclusion, this study underscores that the mental health of displaced children is influenced not only by the experience of trauma symptoms, but also by the quality of their social connections, access to education, and perceived life satisfaction. These findings highlight the importance of addressing both risk and protective factors when designing interventions.

A rights-based, strengths-oriented framework should therefore underpin all psychosocial initiatives, ensuring that children’s dignity, agency, and cultural context are respected and promoted (United Nations, 1989; Ungar, 2011). Programs must go beyond merely alleviating distress to actively fostering resilience, hope, and a sense of belonging, particularly in the world’s most vulnerable and under-resourced regions.

By centering interventions on children’s inherent capacities and aspirations, practitioners and policymakers can better support their recovery and empower them to rebuild meaningful and fulfilling lives.

By situating mental health within a socio-ecological framework, these findings support strength-based and context-sensitive approaches to research and intervention (Bartniczak et al., 2024).

Recent global mental health scholarship has called for greater attention to locally meaningful indicators of well-being and resilience, particularly in underrepresented regions such as the Sahel (Betancourt et al., 2020; Tol et al., 2020; Theron, 2023). The current study responds to this call by empirically demonstrating the relevance of positive mental health indicators in a context characterized by chronic instability and displacement.

## Data Availability

The datasets generated and analyzed during the current study are not publicly available due to the vulnerable status of the child participants and the ethical requirements to protect their confidentiality. Data may be made available from the corresponding author on reasonable request, subject to approval by the Ethics Committee of Niamey’s General Hospital (Niger).
